# Intraoperative mechanical ventilation management in COPD: A state of the art review

**DOI:** 10.1016/j.clinsp.2026.101140

**Published:** 2026-09-10

**Authors:** Jaber S. Alqahtani, Abdulrhman Y. Gazwani

**Affiliations:** a Department of Respiratory Care, Prince Sultan Military College of Health Sciences, Dhahran, Saudi Arabia; b Department of Anesthesia Technology, Prince Sultan Military College of Health Sciences, Dhahran, Saudi Arabia; c College of Medicine and Health, University of Birmingham, Birmingham, United Kingdom

**Keywords:** COPD, Intraoperative, Mechanical ventilation, Lung, Approaches

## Abstract

Chronic Obstructive Pulmonary Disease (COPD) is a prevalent condition in surgical patients and is recognized as a significant risk factor for negative postoperative outcomes. Such adverse outcomes include increased admissions to intensive care units, extended hospitalizations, prolonged ventilation, and elevated rates of perioperative morbidity and mortality. Intraoperative mechanical ventilation is a crucial strategy for managing COPD and improving postoperative outcomes in this high-risk population. However, typical ventilation measures employed during general anesthesia may exacerbate COPD-related physiological dysfunctions if not appropriately optimized. Patients with COPD are particularly susceptible to expiratory flow limitation, dynamic hyperinflation, Intrinsic Positive End-Expiratory Pressure (PEEPi), and Ventilation-Perfusion (V/Q) mismatch during positive-pressure ventilation. Controlled tidal volumes, longer expiratory duration, appropriate external PEEP, and cautious ventilation mode selection are needed to address these pathophysiological concerns. This narrative review synthesizes the current evidence on preoperative assessments relevant to ventilation in patients with COPD, examines intraoperative ventilation strategies, and highlights the underlying physiological principles and clinical implications of various approaches used during surgery. Overall, the available evidence supports adopting a personalized, patient-guided approach to intraoperative ventilation rather than uniform ventilatory targets. Although COPD is a notable risk factor for respiratory and non-respiratory complications in the perioperative context, there is a lack of studies specifically addressing intraoperative ventilation management in COPD. Therefore, there is a pressing need for future research to inform decision-making for personalized intraoperative ventilation strategies and to improve clinical outcomes and prognosis in this most vulnerable population.

## Highlights

• COPD is a prevalent condition among surgical patients and is recognized as a significant risk factor for negative postoperative outcomes.
• Intraoperative mechanical ventilation is a crucial strategy for managing COPD, but it may exacerbate COPD-related physiological dysfunctions if not appropriately optimized.
• The available evidence supports adopting a personalized approach to intraoperative ventilation that is guided by the patient’s physiological parameters, rather than by uniform ventilatory targets.
• Patient‑centered, individualized ventilation plans, integrated within a broader perioperative plan, hold the most promising potential for reducing postoperative complications and improving surgical outcomes in patients with COPD.
• Future studies should focus on COPD-specific research that considers the severity of the disease, phenotypic variability, baseline gas exchange abnormalities, and different ventilation approaches.

## Introduction

Chronic Obstructive Pulmonary Disease (COPD) is one of the significant health challenges in the world and is among the leading causes of morbidity and mortality in the general population.^1^ It is a progressive respiratory disorder impacting around 11% of adults over the age of 25.^2^ Projections indicate a 23.3% increase in COPD cases by 2050, rising from 480 million in 2020 to an estimated 592 million.^2^ The COPD prevalence is still increasing because of the aging of the population, the ongoing exposure to tobacco, and environmental risk factors.^3^ COPD also imposes a vast and rising economic burden, projected to reach tens of trillions of dollars cumulatively by 2050, alongside billions of exacerbations annually.^4^

Patients with COPD are increasingly surviving long enough to receive various elective and emergency surgical procedures. This makes COPD a prevalent condition within surgical populations, particularly in thoracic, upper abdominal, vascular, and orthopedic surgery, where the presence of underlying respiratory disease has a significant effect on the perioperative risk profile.^5^ Large cohort studies indicate that nearly 10% to 20% of adults undergoing major surgery are diagnosed with COPD, with specific reports suggesting that almost one in five surgical patients may be preoperatively impacted.^6^^,^^7^

Inadequate intraoperative management of COPD may result in Postoperative Pulmonary Complications (PPCs), defined as respiratory adverse events occurring in the postoperative period that lead to adverse clinical outcomes.^8^^,^^9^ People living with COPD are vulnerable to PPCs due to advanced age, diminished preoperative Forced Expiratory Volume in one second (FEV_1_), suboptimal functional status, smoking history, emergency surgical procedures, the length and type of anesthesia used, as well as the characteristics of the surgical intervention itself.^10^^,^^11^ Some of the common PPCs include atelectasis, pneumonia, bronchospasm, long-term mechanical ventilation, respiratory failure, and an acute exacerbation of a pre-existing lung disease.^8^^,^^10^ The prevalence of PPCs is significantly greater in patients with COPD compared to those without a chronic lung disease, with established risks associated with surgery and disease severity ranging from two to six times greater.^12^ These complications have a significant clinical and economic impact, such as extended hospital and intensive care unit stay, high cost of healthcare, delayed recovery, and high perioperative mortality.^9^

Intraoperative mechanical ventilation has emerged as a key factor influencing perioperative outcomes in patients with COPD.^9^^,^^13^ The choice of ventilatory settings can significantly impact dynamic hyperinflation, gas trapping, and the likelihood of developing PPCs, either worsening or alleviating these issues.^9^ In conditions like COPD and other flow-limited states, expiratory flow limitation frequently occurs during mechanical ventilation and serves as a significant predictor of PPCs.^10^^,^^14^ This highlights the critical need for tailored, lung-protective strategies.^8^^,^^15^ Improper use of positive-pressure ventilation during general anesthesia significantly modifies respiratory mechanics by decreasing Functional Residual Capacity (FRC), facilitating airway closure, and exacerbating ventilation-perfusion (V/Q) mismatch.^9^^,^^16^ In patients with COPD, these alterations exacerbate expiratory flow limitation and PEEPi, thereby elevating the work of breathing and the risk of developing PPCs unless intraoperative mechanical ventilation is properly optimized.^9^

Thus far, there is no single narrative review that has consolidated the existing evidence on intraoperative mechanical ventilation management in patients with COPD, with the current gap being the absence of COPD-specific guidance on anesthesia practice. Current evidence remains fragmented, diverse and frequently based on non-COPD populations. A coherent, physiology-driven framework is urgently required to inform decision-making for intraoperative mechanical ventilation management, minimize postoperative complications, and enhance perioperative outcomes in this high-risk group.

## Methods

This narrative review was informed by a structured search of PubMed/MEDLINE and Embase from inception to March 2026. Search terms combined the concepts of COPD, general anesthesia, intraoperative or mechanical ventilation, lung-protective ventilation, PEEP, tidal volume, and postoperative pulmonary complications. English-language randomized controlled trials, cohort studies, systematic reviews, physiological studies, and clinical guidelines were eligible; reference lists of key articles were hand-searched for additional sources. Because the aim was conceptual synthesis rather than a quantitative systematic review, no formal risk-of-bias scoring was performed; instead, studies were appraised narratively, with explicit attention to whether evidence was COPD-specific or extrapolated from non-COPD surgical populations. As this paper is a narrative review, PRISMA and other study-specific reporting guidelines are not applicable.

### Pathophysiology of COPD relevant to mechanical ventilation

COPD is a persistent lung disease that leads to permanent airflow obstruction by a combination of mild airway illness, parenchymal damage, and loss of elastic recoil.^17^ These pathological processes cause airway resistance to increase, particularly during expiration, and constitute the basis of the physiology of expiratory flow limitation in individuals with COPD.^17^ The expiration process is primarily passive in the normal state. In COPD, the airways become narrow and collapse during expiration, which affects airflow and limits lung emptying. This restriction is further exacerbated during controlled mechanical ventilation, particularly in cases where the expiratory time is inadequate, making the patient vulnerable to air trapping and progressive lung hyperinflation.^17^

Dynamic hyperinflation occurs when the expiration period is shorter than the time it takes to empty an alveolus. Consequently, this causes the end-expiratory lung volume to rise with each breath, resulting in PEEPi.^18^ Some of the significant implications of PEEPi development during general anesthesia include high work of breathing, high intrathoracic pressure, and compromised venous return, which can result in hypotension and decreased cardiac output.^19^ PEEPi is also a contributor to barotrauma and volutrauma in patients undergoing mechanical ventilation, especially when coupled with high tidal volumes or excessively high respiratory rates.^20^ This is also exacerbated by the effects of anesthetic agents, which reduce respiratory drive, alter chest wall compliance, and inhibit protective airway reflexes.^21^

An additional key pathophysiologic characteristic of COPD, with significant implications for intraoperative management, is V/Q mismatch.^22^ Destruction of alveolar-capillary units and heterogeneous obstruction of the airways result in areas of a low V/Q ratio, which is the primary cause of chronic hypoxemia.^23^ This mismatch can be exacerbated by the use of positive-pressure ventilation and atelectasis induced during general anesthesia, which redistributes ventilation to already well-ventilated lung units, while poorly ventilated areas remain under-ventilated.^24^ Inappropriate ventilatory settings may thus improve global oxygenation at the expense of regional overdistension and lung injury.^24^

**Fig. 1:**
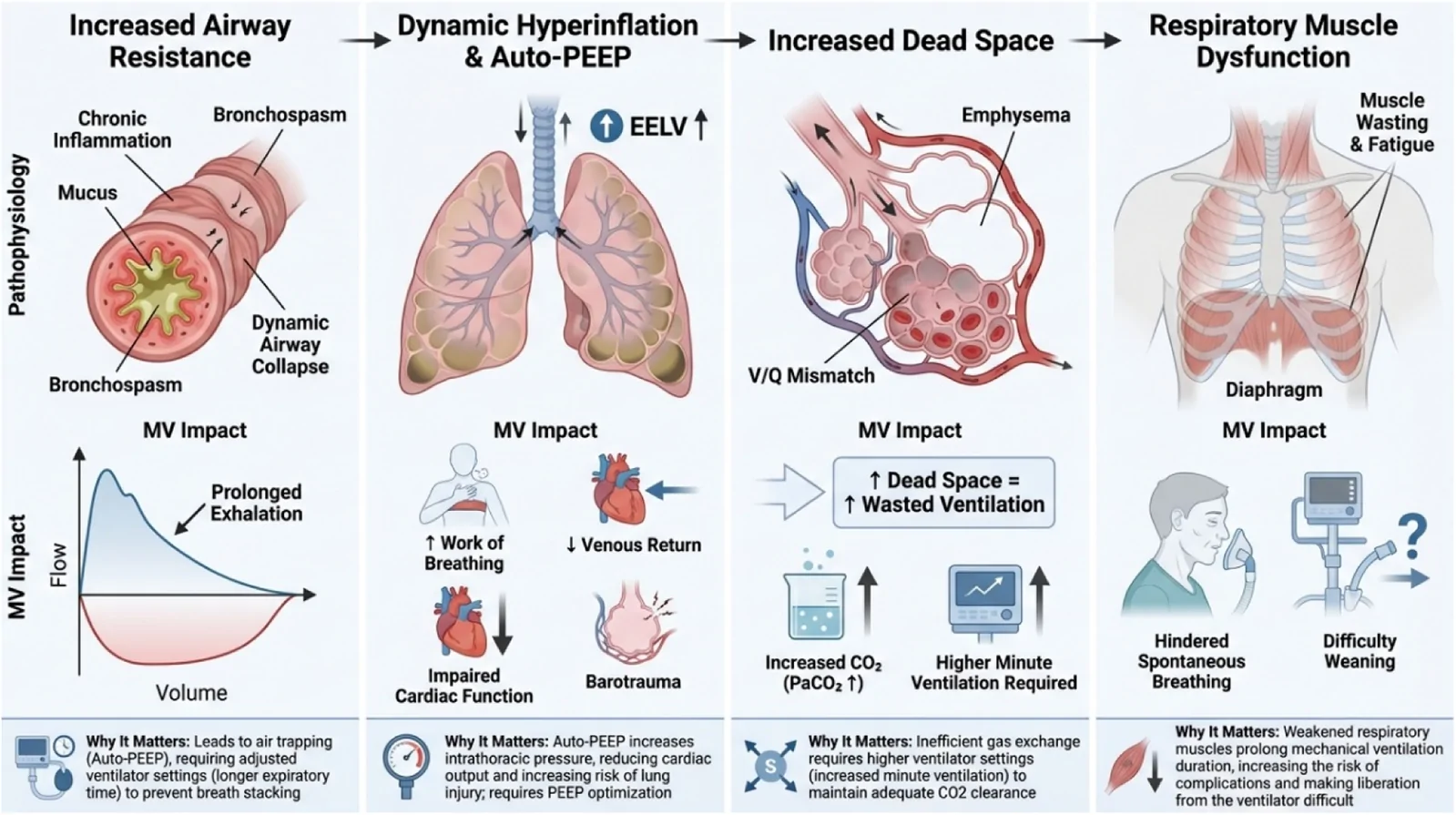
Pathophysiology of COPD relevant to mechanical ventilation. MV, Mechanical Ventilation; PEEP, Positive End-Expiratory Pressure; Auto-PEEP, Auto-Positive End-Expiratory Pressure; EELV, End-Expiratory Lung Volume; V/Q, Ventilation/Perfusion; PaCO_2_, Partial Pressure of Carbon dioxide in arterial blood; CO₂, Carbon Dioxide.

These pathophysiological changes predispose COPD patients to the undesirable consequences of mechanical ventilation during general anesthesia. Therefore, understanding the mechanisms is essential for effectively applying physiological principles to ensure safe and efficient intraoperative mechanical ventilation techniques (Fig. 1). This knowledge will help minimize postoperative pulmonary and non-pulmonary complications, providing optimal gas exchange.

### Preoperative assessment relevant to mechanical ventilation in COPD

The preoperative evaluation is crucial for identifying patients with COPD who are at risk of postoperative complications during the perioperative period, as well as for establishing intraoperative ventilation goals.^25^ The assessment begins with the evaluation of COPD severity, which is typically assessed using the Global Initiative for Chronic Obstructive Lung Disease (GOLD) criteria, including airflow limitation (spirometry), symptom load, exacerbation history, and multimorbidity profile.^26^ In addition, clinical phenotype identification, such as emphysema-dominant versus chronic bronchitis-dominant disease, provides more information on lung mechanics, gas-exchange defects, and susceptibility to dynamic hyperinflation during mechanical ventilation.^27^

The focus of preoperative assessment has been on the Pulmonary Function Tests (PFTs). The FEV_1_, Forced Vital Capacity (FVC), and the ratio of FEV_1_ to FVC have been objective measures of airflow obstruction.^26^ A decreased preoperative FEV_1_ is also associated with an increased risk of PPCs.^28^ Lung volumes and diffusion capacity can also be used, when possible, to recognize patients at risk of air trapping and dysfunctional gas exchange.^25^ Arterial blood gas analysis is also required in patients with more severe illness because a baseline of hypercapnia is a sign of chronic ventilatory defects and low respiratory capacity.^29^ Chronic retention of carbon dioxide should be recognized to prevent excessive vigorous ventilation of the patient in the operating room, which can lead to respiratory alkalosis or exacerbate dynamic hyperinflation.^29^

**Fig. 2:**
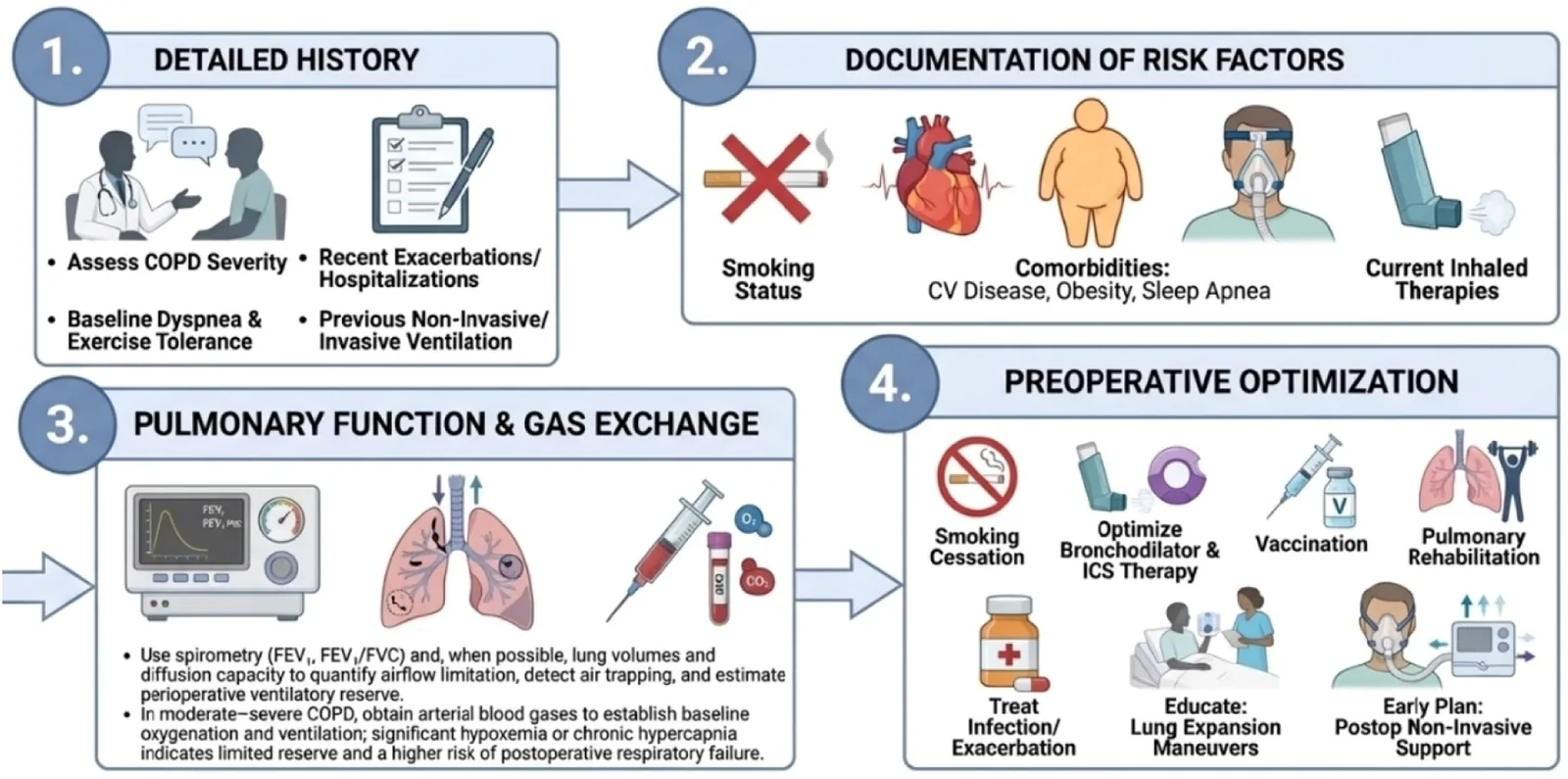
Preoperative assessment relevant to ventilation in COPD. COPD, Chronic Obstructive Pulmonary Disease; CV, Cardiovascular; FEV_1_, Forced Expiratory Volume in one second; FVC, Forced Vital Capacity; ICS, Inhaled Corticosteroid; O_2_, Oxygen; CO_2_, Carbon dioxide.

One crucial factor in perioperative risk reduction is preoperative optimization of respiratory status.^25^ Research involving people living with COPD revealed that administering inhaled bronchodilators and steroids for 10-days before surgery enhanced extubation times and reduced postoperative hospital stays.^30^ When possible, acute respiratory infections and COPD exacerbations must be treated, and elective surgery should be postponed.^31^ Even smoking cessation shortly before surgery has been found to enhance mucociliary activity and decrease respiratory complications after surgery.^32^^,^^33^ Fig. 2 presents a summary of preoperative assessment relevant to ventilation in COPD.

### Intraoperative mechanical ventilation strategies in COPD: core principles and evidence

COPD patients are more prone to ventilator-induced lung damage, so intraoperative mechanical ventilation needs to be informed by disease-specific respiratory mechanics.^34^ The aim is to optimize oxygenation and carbon dioxide elimination, while reducing dynamic hyperinflation, auto-PEEP, and airway pressures.

It must be emphasized that most of the targets described below are extrapolated from lung-protective ventilation trials in mixed surgical and critically ill populations rather than derived from COPD-specific randomized data; they should therefore be regarded as physiologically rational and reasonable defaults rather than evidence-proven thresholds.^35^^,^^36^

Excessive minute ventilation should be avoided during intraoperative mechanical ventilation in patients with COPD. Aggressive treatment of hypercapnia by increasing tidal volume or respiratory rate can raise the extent of expiratory flow limitation and air trapping.^37^

Ventilatory behavior is strongly phenotype-dependent. Emphysema-predominant patients exhibit greater loss of elastic recoil and are most prone to dynamic hyperinflation, favoring lower respiratory rates, prolonged expiratory time, and cautious external PEEP, while chronic-bronchitis-predominant patients may tolerate modestly higher PEEP.^27^ Obesity-associated COPD and overlap syndrome shift the balance toward atelectasis and may benefit from individualized PEEP titration, whereas coexisting pulmonary hypertension or chronic hypercapnia warrants particular caution with permissive hypercapnia and high airway pressures. Ventilation should therefore be tailored to the dominant phenotype rather than applied uniformly.^18^^,^^27^

The method of ventilating during general anesthesia is a modifiable factor that can significantly reduce postoperative adverse outcomes. Currently, the leading intraoperative ventilation modes include Volume-Controlled Ventilation (VCV), Pressure-Controlled Ventilation (PCV), and Pressure-Controlled Ventilation with Volume Guarantee (PCV-VG). In comparison to VCV, PCV has been shown to offer enhanced oxygenation, reduced peak inspiratory pressure, decreased release of inflammatory mediators, and improved dynamic compliance in patients undergoing thoracic and abdominal surgeries.^38^, ^39^, ^40^, ^41^, ^42^ On the other hand, an observational study involving more than 100,000 patients indicated that the incidence of PPCs was elevated in the PCV group compared to the VCV group.^43^ Considering the features of both PCV and VCV, it was proposed that PCV-VG could reduce airway pressure and enhance oxygenation.^44^^,^^45^ To date, there is a lack of conclusive evidence indicating that any specific ventilation mode is superior to others in minimizing the risk of PPCs in COPD.^36^ However, VCV can provide a steady minute ventilation and predictable carbon dioxide elimination, while PCV could also improve the distribution of gases and peak airway pressures in patients with heterogeneous lung disease when the inspiratory flow is decelerated.^36^ Therefore, the selection of the mode relies on the preferences of the ventilation team and the needs of the patients.

The COPD ventilation safety depends on the choice of tidal volume. The lungs of patients with COPD are usually overinflated; however, large tidal volumes can raise auto-PEEP and dynamic hyperinflation. According to perioperative and critical care literature, low to moderate tidal volumes, at 6‒8 mL/kg of estimated body weight, minimize volutrauma and barotrauma.^35^ COPD-specific randomized controlled trials are limited; however, evidence from lung-protective ventilation practices suggests that a conservative tidal volume can offset mechanical stress on vulnerable lung units without compromising gas exchange in patients, based on patient characteristics.^35^

Contemporary lung-protective frameworks increasingly emphasize driving pressure, mechanical power, and transpulmonary pressure rather than tidal volume alone. Lower intraoperative driving pressure (plateau pressure minus PEEP) is associated with fewer PPCs.^46^ Mechanical power integrates the cumulative energy delivered to the lung, and esophageal-pressure-derived transpulmonary pressure can help separate lung from chest-wall mechanics.^47^ Although COPD-specific validation of these targets is lacking and dynamic hyperinflation may distort plateau-pressure interpretation, minimizing driving pressure and mechanical power is a physiologically coherent goal that warrants prospective study in this population.^46^^,^^47^

In COPD, the presence of auto-PEEP is prevalent, resulting in higher work of breathing and compromised hemodynamics.^17^ Therefore, it is essential to consider external PEEP alongside auto-PEEP for better intraoperative ventilation management. In some patients, low external PEEP may partially reverse auto-PEEP and reduce inspiratory threshold load, as well as enhance patient-ventilator synchronization.^48^ To prevent any exacerbation of hyperinflation or circulatory compromise, it’s essential to maintain the external PEEP below 75% to 85% of the auto-PEEP.^17^ This 75%–85% relationship should be understood as a physiological principle rather than a routinely achievable bedside target. This is because intrinsic PEEP is seldom measured in the operating room and reliable quantification requires an end-expiratory occlusion under fully passive, controlled conditions. Many anesthesia ventilators do not facilitate this measurement, and the value becomes unreliable during spontaneous or partially assisted breathing. In practice, external PEEP is therefore titrated empirically while monitoring airway pressures, expiratory flow curves, and hemodynamics.^49^^,^^50^ A meta-analysis conducted in 2013, which included eight randomized controlled trials, revealed that employing intraoperative PEEP levels ranging from 3 to 12 cm H_2_O significantly reduced the risk of developing PPCs (relative risk, 0.29; 0.14 to 0.60).^51^ Excessive external PEEP is to be avoided, as it raises the end-expiratory lung volume and aggravates hyperinflation, particularly in patients with severe airflow obstruction.^52^

To avoid dynamic hyperinflation during intraoperative mechanical ventilation in patients with COPD, it's essential to fine-tune ventilator settings. This can be achieved by reducing the respiratory rate and extending the expiratory phase, allowing for sufficient time for exhalation. Lowering the respiratory rate and adopting an inspiratory-to-expiratory ratio of at least 1:3 or longer in cases of severe airflow limitation would promote greater lung emptying, which in turn reduces air trapping and PEEPi.^53^^,^^54^

**Fig. 3:**
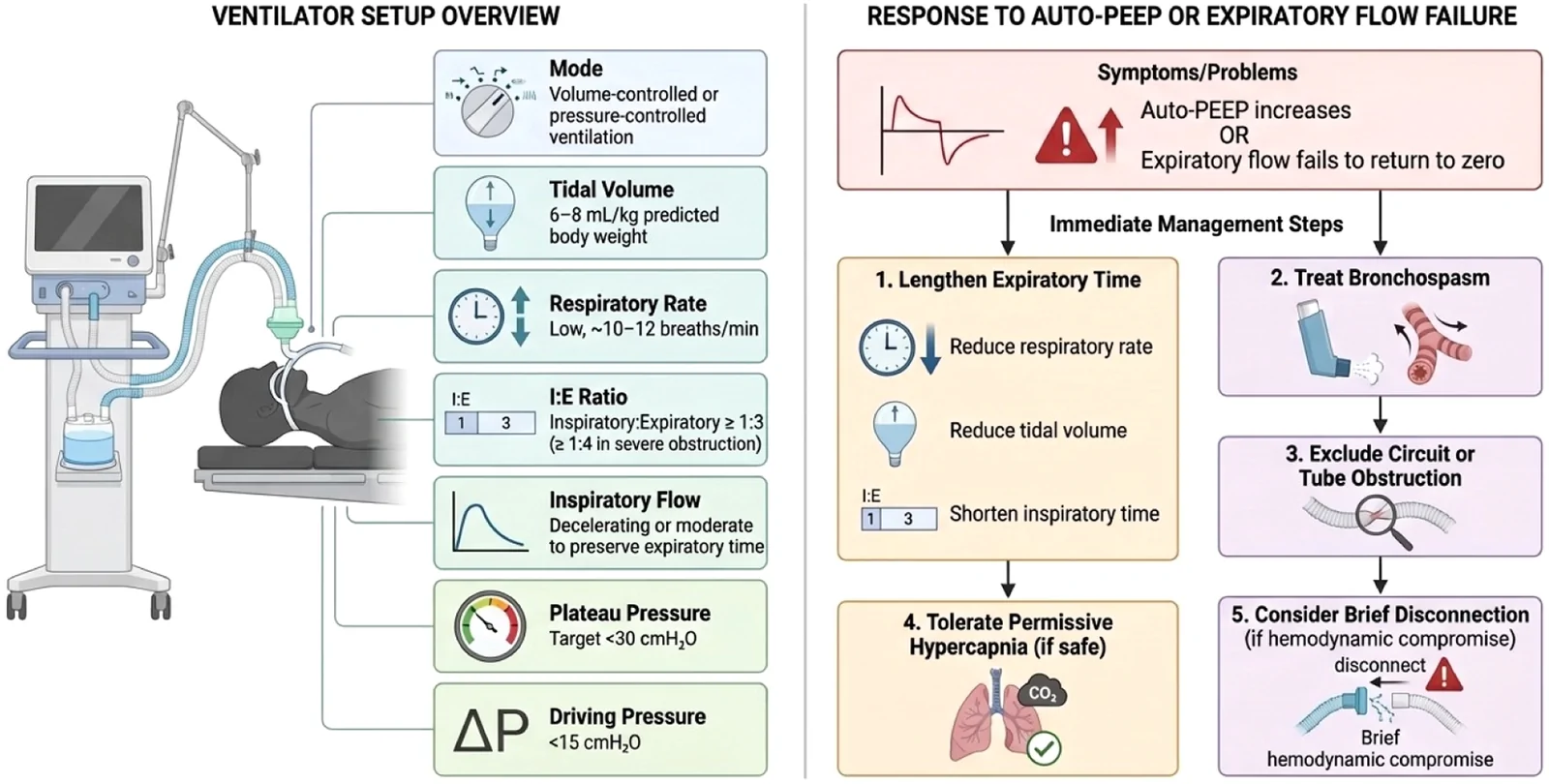
Proposed practical intraoperative ventilator setup and management of common COPD ventilation issues. PEEP, Positive End-Expiratory Pressure; Auto-PEEP, Auto-Positive End-Expiratory Pressure; I:E, Inspiratory-to-Expiratory ratio; CO_2_, Carbon Dioxide; cmH_2_O, Centimeters of water pressure.

Regarding practical intraoperative ventilator setup, we suggest some pragmatic starting points to be individualized for each patient (see Fig. 3). Such initial settings include mode selection, tidal volume range, RR, inspiratory-to-expiratory ratio, inspiratory flow, plateau pressure target and driving pressure limit.^17^^,^^53^ Furthermore, Fig. 3 shows some troubleshooting approaches to rising auto-PEEP or dynamic hyperinflation.

Moreover, personalized ventilation in COPD is facilitated by close intraoperative monitoring. Inspection of the expiratory flow-time curve for failure of flow to return to baseline provides an immediate, ventilator-based signal of incomplete emptying and dynamic hyperinflation, while capnography waveform analysis reflects expiratory flow limitation and V/Q changes.^55^^,^^56^ Continuous measurement of dynamic compliance and driving pressure helps detect evolving hyperinflation or derecruitment.^55^ Further, electrical impedance tomography for regional ventilation distribution and esophageal manometry for transpulmonary pressure can further individualize PEEP and tidal volume where available.^57^^,^^58^

Lung recruitment maneuvers do not have a significant influence on intraoperative ventilation in COPD, where the primary issue is hyperinflation rather than merely a loss of aeration.^34^ Reopening of atelectatic regions can have a short-term beneficial effect on ventilation; however, it is also likely to cause overdistension and hemodynamic dysfunction when applied to hyperinflated lungs.^34^ Because COPD is characterized by markedly heterogeneous regional time constants, an applied recruitment pressure is distributed unevenly.^59^ Thus, relatively healthy or already-hyperinflated emphysematous units receive a disproportionate share of the pressure and are at risk of regional overdistension and barotrauma, while truly collapsed regions may remain unrecruited. The net effect can be worsening dynamic hyperinflation rather than improved aeration.^60^ In addition, the sustained rise in intrathoracic pressure reduces venous return and right ventricular preload, which may precipitate hypotension and reduced cardiac output, particularly in patients with limited cardiovascular reserve.^61^

**Fig. 4:**
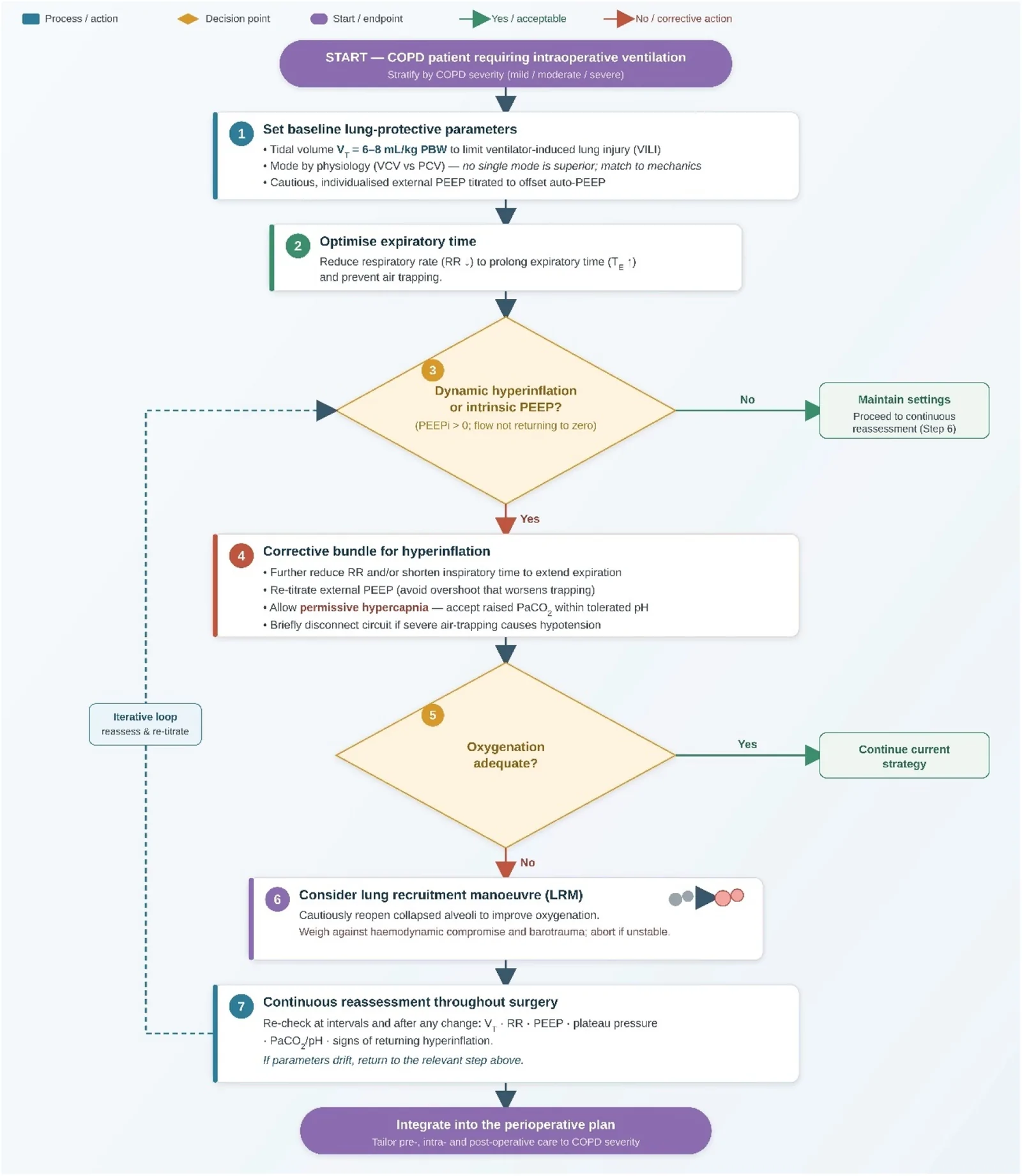
Proposed algorithm for intraoperative mechanical ventilation strategies in COPD. COPD, Chronic Obstructive Pulmonary Disease; PEEP, Positive End-Expiratory Pressure; Auto-PEEP, Auto-Positive End-Expiratory Pressure; VCV, Volume-Controlled Ventilation; PCV, Pressure-Controlled Ventilation; VILI, Ventilator-Induced Lung Injury; LRM, Lung Recruitment Manoeuvre; PaCO_2_, Arterial Carbon Dioxide Pressure; PBW, Predicted Body Weight.

Given the limited availability of robust COPD-specific data, experts advise that recruitment in this population should be approached with caution and utilized only when there is a clear indication for refractory atelectasis. This should involve brief, targeted maneuvers that are closely monitored for airway pressures, oxygenation, and cardiovascular responses. Fig. 4 illustrates the recommended intraoperative ventilation strategies for patients with COPD.

To sum up, implementing protective lung strategies (appropriate tidal volume, level of PEEP, and use of lung recruitment maneuvers) is essential for ensuring safer intraoperative mechanical ventilation in COPD.^62^ These strategies effectively minimize ventilator-induced lung injury and help control dynamic hyperinflation.

### Comparative outcomes: volume-controlled versus pressure-controlled ventilation

The debate over whether VCV or PCV is preferable during general anesthesia in patients with COPD has intensified due to the distinct mechanical and physiological characteristics of each mode. VCV is commonly used during surgical operations and delivers a consistent volume of tidal volume on each breath, resulting in consistent minute ventilation and carbon dioxide elimination.^63^ However, when airway resistance and expiratory flow are impaired, as in COPD, VCV may be associated with increased peak airway pressure and a higher likelihood of dynamic hyperinflation when expiratory time is short.^53^^,^^64^ This may lead to barotrauma and unfavorable hemodynamic outcomes, particularly among patients with severe airflow obstruction.^64^^,^^65^

On the other hand, PCV regulates inspiratory pressure, resulting in variations in delivered tidal volume based on the compliance and resistance of the patient's lungs and airways.^66^ The decelerating, relatively slow inspiratory flow pattern characteristic of PCV may enhance gas distribution uniformity and reduce peak airway pressures, which could be beneficial for patients with heterogeneous lung conditions, such as COPD.^66^^,^^67^ Studies have found a decreased peak inspiratory pressure in PCV than VCV, while improvements in oxygenation and clearance of carbon dioxide are inconsistent.^64^ Variation in delivered tidal volume on PCV requires close follow-up, as unexpected changes in respiratory muscle function or surgical circumstances may result in hypoventilation or hypercapnia.^64^^,^^68^

Importantly, the apparent benefits of PCV are derived almost exclusively from physiological endpoints, such as peak airway pressure, oxygenation, and dynamic compliance, in predominantly non-COPD surgical populations. Also, such outcomes have not translated into demonstrable superiority in clinically meaningful outcomes, as no ventilation mode has been shown to reduce postoperative pulmonary complications, length of stay, or mortality in COPD.^36^^,^^43^

There is limited clinical outcome data on the use of VCV versus PCV in patients with COPD, and the few existing studies have not yielded significantly different results regarding the incidence of postoperative adverse outcomes, length of stay, and mortality.^36^ Consequently, the existing evidence does not justify a consistent preference towards one mode over the other. Instead, the ventilation mode should be chosen based on the patient's specific physiology, intraoperative respiratory mechanics, and the clinician's ability to closely monitor and control ventilatory parameters. A personalized, physiology‑based intervention is essential for optimizing outcomes in this population.

### Implications of intraoperative mechanical ventilation strategies on postoperative outcomes in COPD patients

PPCs are one of the leading causes contributing to the increased burden of postoperative outcomes in patients with COPD undergoing surgery. Postoperative respiratory events that impede recovery and clinical outcomes are known as PPCs. Typical forms of PPCs include atelectasis, pneumonia, bronchospasm, acute respiratory failure, prolonged mechanical ventilation, and acute exacerbations of COPD.^10^ Individuals diagnosed with COPD experience more adverse outcomes (longer hospital stay and prolonged mechanical ventilation use) related to PPCs compared to those without the condition.^13^

Epidemiological studies indicate that COPD is among the most robust independent predictors of PPCs across multiple surgical procedures. The prevalence of PPCs was notably higher in individuals with COPD, standing at 26.7%, in contrast to 10.2% in those without such a history (p < 0.001).^69^ Such PPCs elevate both the clinical and financial burdens on the health system by driving up healthcare utilization and costs.^70^

**Fig. 5:**
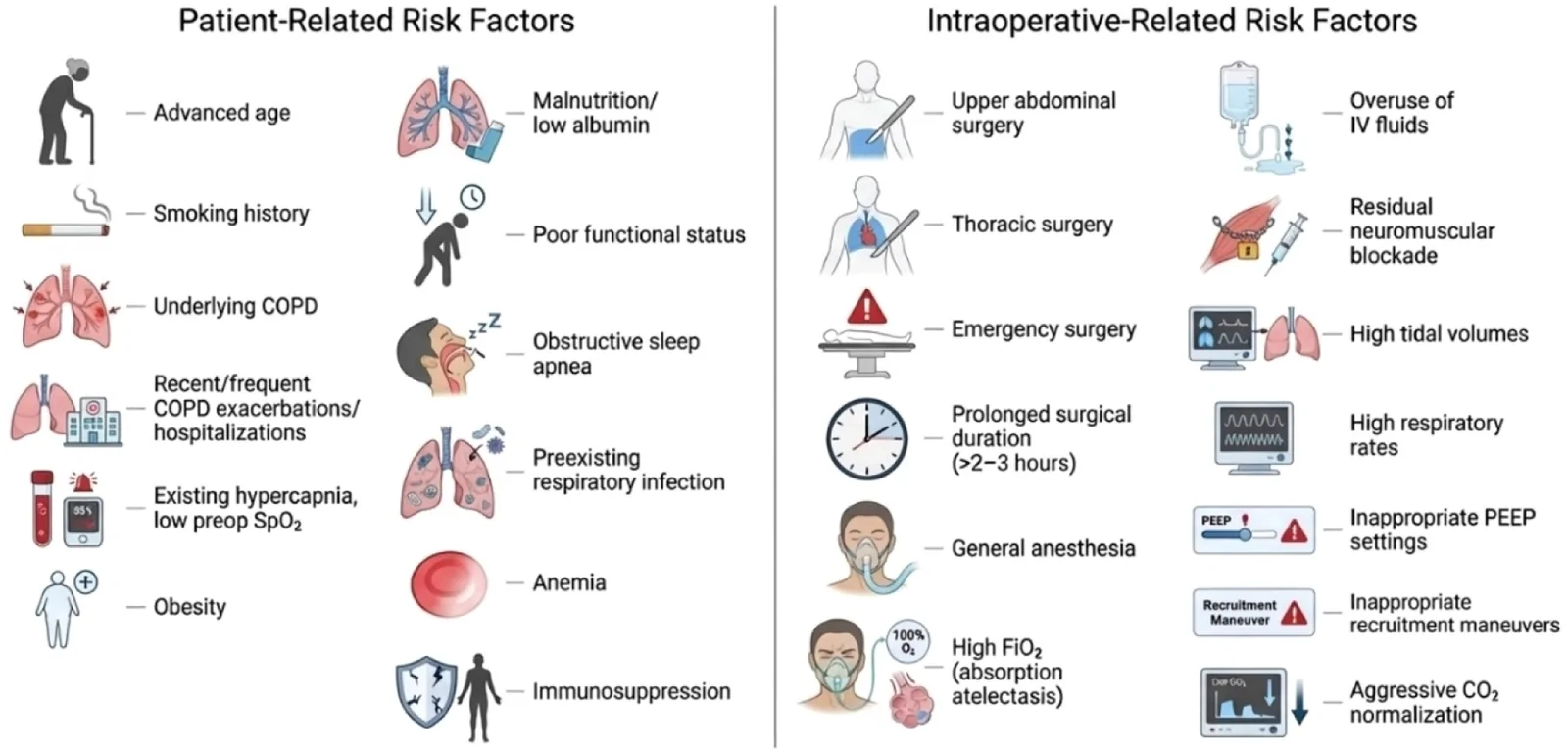
Risk factors for Postoperative Pulmonary Complications (PPCs). COPD, Chronic Obstructive Pulmonary Disease; SpO_2_, Peripheral Oxygen Saturation; FiO_2_, Fraction of Inspired Oxygen; PEEP, Positive End-Expiratory Pressure; CO_2_, Carbon Dioxide.

In COPD, the risk of PPCs is influenced by patient characteristics, the nature of the surgical procedure, and modified perioperative management strategies.^10^^,^^25^^,^^71^ Risks associated with patients encompass factors such as advanced age, recent or frequent exacerbations or hospitalizations due to respiratory issues, existing hypercapnia, and low preoperative SpO_2_ levels. Additionally, current tobacco use and comorbidities like cardiovascular disease, obesity, and inadequate nutritional status are significant factors to consider.^25^^,^^72^^,^^73^ Thoracic and upper abdominal surgeries are associated with notably elevated rates of PPCs due to their impact on diaphragmatic function, lung volumes, and mucociliary clearance (Fig. 5). Factors such as emergency procedures, general anesthesia, and extended operating durations (typically exceeding 2–3 hours) further heighten the risk of pneumonia, respiratory failure, and other complications in patients with COPD.^9^^,^^10^^,^^74^^,^^75^

Specific surgical contexts modify these principles. Laparoscopic and robotic procedures requiring pneumoperitoneum, often combined with steep Trendelenburg positioning, reduce FRC, raise airway and plateau pressures, and promote atelectasis, frequently necessitating modest increases in PEEP and careful plateau-pressure monitoring.^55^ Thoracic surgery and one-lung ventilation are especially demanding in COPD, where the combination of an emphysematous dependent lung, hyperinflation, and hypoxic pulmonary vasoconstriction heightens the risk of both hypoxemia and ventilator-induced lung injury, mandating protective tidal volumes, judicious PEEP, and vigilant gas-exchange monitoring.^55^^,^^76^

There are a significant number of intraoperative parameters that are adjustable and influenced by healthcare provider experience.^34^^,^^77^ Respiratory dysfunction following surgery can be worsened by inappropriate ventilatory settings that lead to dynamic hyperinflation, elevated airway pressures, or atelectasis, consequently increasing the likelihood of PPCs.^9^^,^^34^^,^^77^ Inadequate management of pain during and following surgery, overuse of intravenous fluids, and residual effects of neuromuscular blockade can negatively affect ventilation and the ability to cough effectively, increasing the risk of secretion retention, atelectasis, and pneumonia.^9^^,^^78^, ^79^, ^80^ These parameters are, nonetheless, adjustable and underscore the importance of respiratory management during and immediately after surgery in minimizing PPCs and improving outcomes for patients with COPD.

Optimal perioperative respiratory care extends beyond the intraoperative period. In high-risk and COPD patients, prophylactic or early postoperative noninvasive ventilation and high-flow nasal oxygen can reduce reintubation and postoperative respiratory failure, and selected patients may be extubated directly to noninvasive respiratory support.^81^ Postoperative chest physiotherapy, early awake mobilization, adequate analgesia to preserve cough, and enhanced recovery after surgery pathways further reduce pulmonary complications and should be integrated with intraoperative lung-protective ventilation as part of a comprehensive perioperative bundle.^82^, ^83^, ^84^

On the other hand, people living with COPD are at an increased risk for significant postoperative non-pulmonary complications, including stroke, acute kidney injury, and wound infection.^85^ These issues are indicative of systemic inflammation, diminished physiological reserve, and the prevalence of cardiovascular comorbidities.^85^ These outcomes reflect the systemic nature of COPD.^86^ Chronic low-grade systemic inflammation contributes to endothelial and cardiovascular dysfunction, while frailty, skeletal-muscle dysfunction and sarcopenia, and nutritional impairment reduce physiological reserve and impair postoperative recovery, weaning, and rehabilitation.^86^^,^^87^ Recognizing and, where possible, optimizing these systemic traits preoperatively through nutritional support, prehabilitation, and cardiovascular risk management is therefore an integral part of perioperative care in COPD.^87^^,^^88^

In a study involving more than 22,000 patients with COPD who underwent different surgical procedures, it was found that postoperative non-pulmonary complications such as cardiac events, sepsis, and renal dysfunction played a significant role in extending hospital stays, increasing ICU admissions, and raising in-hospital mortality rates when compared to matched controls without COPD.^89^ In another study, patients with COPD demonstrated a significantly higher incidence of various postoperative complications.^90^ Although in‑hospital mortality was increased among individuals with GOLD 3 COPD, disease severity did not influence the occurrence of other postoperative complications.^90^ Indeed, COPD is a strong predictor of postoperative mortality and morbidity; thus, it is critical to enhance perioperative management for these patients to reduce the significant health and economic burden on healthcare systems.^89^

### Recommendations for intraoperative ventilation management in patients with COPD

The following recommendations rest predominantly on physiological rationale and evidence extrapolated from non-COPD surgical populations rather than on COPD-specific randomized trials, and should be applied with corresponding clinical judgment. Physiological principles, current available evidence, and ongoing clinical assessment should guide adequate intraoperative ventilation for patients with COPD, rather than adhering to fixed compliance with standard ventilatory goals. Preventing dynamic hyperinflation and PEEPi, while maintaining sufficient oxygenation and acceptable carbon dioxide levels, should be the central focus of ventilation strategies in COPD patients.^91^ Tidal volumes of 6‒8 mL/kg of predicted body weight are supported to minimize high airway pressures and reduce the occurrence of volutrauma and barotrauma.^92^ The respiratory rates must also be adjusted to allow sufficient expiratory time, with long inspiratory-to-expiratory ratios often necessary to reduce air trapping.

External PEEP needs to be personalized and used with precaution. Low basal levels of external PEEP can be beneficial in countering intrinsic PEEP and enhancing ventilatory efficiency in the selected patients. However, excessive PEEP must be avoided because it increases the risk of hyperinflation and hemodynamic compromise.^49^ Closely monitoring for airway pressures, capnography, and hemodynamic parameters is necessary to inform the process of titrating PEEP. VCV and PCV can be safely applied in COPD patients, provided that ventilatory settings are continuously reviewed and adjusted according to changes in respiratory mechanics and surgical scenarios.

When necessary, permissive hypercapnia should be considered, as aggressive attempts to normalize arterial carbon dioxide can worsen expiratory flow limitation and lead to dynamic hyperinflation.^53^^,^^93^ However, permissive hypercapnia is not universally appropriate. The accompanying respiratory acidosis raises pulmonary vascular resistance and may worsen pre-existing pulmonary hypertension and right ventricular dysfunction.^94^ Also, it can increase intracranial pressure, provoke sympathetic activation with arrhythmia or hemodynamic instability, and confound postoperative neurologic assessment. It should therefore be applied cautiously and generally avoided in patients with raised intracranial pressure, significant pulmonary hypertension or right ventricular failure, or hemodynamic instability.^54^^,^^94^ In general, intraoperative ventilation, which is individualized and physiology-guided as part of a larger perioperative plan, is a key focus for reducing postoperative adverse events and improving patient outcomes in patients with COPD undergoing surgery.

### Gaps in evidence and future directions

Although there has been increased awareness of the need for optimal intraoperative mechanical ventilation management in patients with COPD, a significant evidence gap remains. There is little evidence from high-quality randomized controlled trials and observation studies specifically targeting COPD populations in the perioperative setting. Much of contemporary practice is based on extrapolations from trials conducted in mixed populations of surgical patients or intubated patients with no explicit chronic airflow limitation. The differences in study designs, ventilatory protocols, and outcome definitions also reduce the generalizability of the current findings, making it difficult to compare the results of the studies directly. Currently, there is a lack of a universally recognized, evidence-based framework that combines preoperative optimization, intraoperative ventilator settings, and postoperative weaning strategies tailored for surgical patients with COPD.

In particular, priorities include randomized trials that are phenotype-stratified (e.g., emphysema-versus chronic-bronchitis-predominant COPD) comparing individualized, physiology-guided ventilation with standard care. Additionally, we require trials that employ driving pressure or mechanical power as physiological endpoints in conjunction with a standardized composite of PPCs. Likewise, prospective perioperative registries specific to COPD are an unmet research need to capture real-world practice and outcomes.

Future studies should focus on COPD-specific research that considers disease severity, phenotypic variability, baseline gas exchange abnormalities, and different ventilation approaches. Pulmonary outcome measures during perioperative care would be better defined through standardized methods, enabling more accurate interpretation of findings and the synthesis of evidence. The development of monitoring technologies and personalized mechanical ventilation techniques, such as physiology-based and phenotype-based strategies, offers a promising new direction in optimizing intraoperative mechanical ventilation. A more robust evidence base is needed to support the development of personalized ventilation plans that balance lung-protective ventilation with effective gas exchange in this high-risk surgical population.

## Conclusion

COPD serves as a significant predisposing risk factor for adverse outcomes during the perioperative period, presenting one of the most pressing challenges in the care of patients undergoing surgery. Alterations in respiratory mechanics commonly observed in COPD, including expiratory flow limitation, dynamic hyperinflation, intrinsic PEEP, and V/Q mismatch, render these patients particularly vulnerable to the adverse effects of general anesthesia and positive-pressure ventilation. In this context, intraoperative mechanical ventilation becomes a crucial and modifiable factor influencing postoperative lung outcomes. This narrative review examines the significance of intraoperative ventilation strategies informed by physiological principles that can effectively tackle the underlying pathophysiology of COPD. Evidence supports the implementation of low-to-moderate tidal volumes, careful control of the respiratory rate to ensure adequate expiration, and personalized application of external PEEP and ventilation mode.

The normalization of arterial carbon dioxide levels should be approached with caution, as aggressive measures may increase the risk of dynamic hyperinflation and ventilator-induced lung injury in patients with COPD. Permissive hypercapnia could be appropriate for certain patients. During surgery, ventilatory parameters must be continuously reevaluated due to the dynamic nature of respiratory mechanics. Although the existing recommendations are based on general perioperative data, critical care research, and fundamental physiological concepts, there is a lack of high-quality evidence specific to COPD. When integrated within a broader perioperative plan, individualized ventilation offers the most promising route to reducing postoperative complications and improving surgical outcomes in COPD.

## Authors’ contributionse

JA and AG: Conceptualization; data curation; formal analysis; investigation; methodology; project administration; resources; validation; visualization; writing-original draft; writing-review & editing.

## Ethics committee information

NA.

## Informed consent and patient details

NA.

## Funding

The author declare that no financial support was received for the research and/or publication of this article.

## Data availability

The original contributions presented in this study are included in the article. Further inquiries can be directed to the corresponding author.

## Declaration of competing interest

The author declare that the research was conducted in the absence of any commercial or financial relationships that could be construed as a potential conflict of interest.
